# Did Nirsevimab Shift Pediatric Hospitalizations Due to Lower Respiratory Tract Infections? A Nationwide Italian Study (2024–2025)

**DOI:** 10.3390/v18030274

**Published:** 2026-02-24

**Authors:** Paolo Manzoni, Matteo Riccò, Chryssoula Tzialla, Graziano Barera, Paolo Del Barba, Simona De Franco, Guido Pellegrini, Enrico Crapanzano, Giangiacomo Nicolini, Andrea Alba, Stefano Fiocchi, Mauro Vivalda, Giulia Natta, Alessandra Casati, Mariano Manzionna, Simone Rugolotto, Laura Saggioro, Simona Pesce, Maria Scavone, Antonietta Distilo, Vincenza Roseto, Antonino Di Toro, Luca Pierri, Gianfranco Scarpelli, Elvira Bonanno, Lidia Decembrino, Enrico Felici, Camilla Selvatico, Valentina Saracco, Francesco Morrone, Claudio Costantino, Cecilia Nobili, Mario Giuffrè

**Affiliations:** 1 SCDU Pediatria e Neonatologia Ospedale Degli Infermi, ASL Biella, 13875 Ponderano, Italy; enrico.crapanzano@aslbi.piemonte.it; 2 Postgraduate School of Pediatrics, University of Turin, 10124 Turin, Italy; cecilia.nobili@unito.it; 3 Servizio di Prevenzione e Sicurezza Negli Ambienti di Lavoro (SPSAL), AUSL-IRCCS di Reggio Emilia, 42123 Reggio Emilia, Italy; 4 SC Pediatria e Nido, ASST Pavia-Ospedale Civile di Voghera, 27058 Voghera, Italy; chryssoula.tzialla@unipv.it; 5 SC Patologia Neonatale, ASST Papa Giovanni XXIII, 24127 Bergamo, Italy; 6 U.O. Pediatria e U.O. Patologia Neonatale, Dipartimento Materno Infantile, IRCCS Ospedale San Raffaele, 20132 Milan, Italy; barera.graziano@hsr.it; 7 U.O. Pediatria, Dipartimento Materno Infantile, IRCCS Ospedale San Raffaele, 20132 Milan, Italy; delbarba.paolo@hsr.it; 8 SC Pediatria e Neonatologia, Dipartimento Materno Infantile, ASL Novara, 28021 Borgomanero, Italy; simona.defranco@asl.novara.it; 9 SC Pediatria e Neonatologia, Dipartimento Materno Infantile, ASST Nord Milano, Sesto San Giovanni, 20099 Milan, Italy; guido.pellegrini@asst-nordmilano.it; 10 UOC Pediatria, Dipartimento Materno Infantile, AULSS 2 “Marca Trevigiana”-Ospedale di Conegliano, 31015 Conegliano, Italy; giangiacomo.nicolini@aulss2.veneto.it; 11 Dipartimento di Salute della Donna e del Bambino, Azienda Ospedaliera Universitaria Padova, 35128 Padova, Italy; andrea.alba@unipd.it; 12 SC Pediatria, Dipartimento Materno Infantile, ASST Ovest Milanese, Magenta, 20013 Milan, Italy; stefano.fiocchi@asst-ovestmi.it; 13 SC Pediatria Carmagnola e Moncalieri e Neonatologia Aziendale, Dipartimento Materno Infantile, ASL Torino 5, 10024 Moncalieri, Italy; vivalda.mauro@aslto5.piemonte.it (M.V.); natta.giulia@aslto5.piemonte.it (G.N.); 14 U.O. Pediatria e Neonatologia, Dipartimento Materno Infantile, ASL Verbania Cusio Ossola, 28921 Verbania, Italy; alessandra.casati@aslvco.it; 15 UOC Pediatria e Neonatologia, Dipartimento di Medicina Dell’età Evolutiva, ASL Bari, 70124 Bari, Italy; mariano.manzionna@asl.bari.it; 16 UOC Pediatria, Dipartimento Materno Infantile, AULSS 5 Polesana, 45100 Rovigo, Italy; simone.rugolotto@aulss5.veneto.it (S.R.); laura.saggioro@aulss5.veneto.it (L.S.); 17 Department of Neonatology and NICU, San Carlo Hospital, 85100 Potenza, Italy; simona.pesce@ospedalesancarlo.it (S.P.); maria.scavone@ospedalesancarlo.it (M.S.); 18 UO di Pediatria e Nido di Paola/Cetraro, Dipartimento Materno Infantile, Spoke Hospital of Cetraro, Azienda Sanitaria Provinciale (ASP) Cosenza, 87022 Cosenza, Italy; a.distilo@aocs.it; 19 UOC-TIN, Ospedale Evangelico Betania, 80147 Naples, Italy; vincenza.roseto@betaniahospital.org; 20 UO Terapia Intensiva Neonatale e Neonatologia, Dipartimento Area Critica, AORN Santobono Pausilipon, 80129 Naples, Italy; a.ditoro@santobonopausilipon.it (A.D.T.); l.pierri@santobonopausilipon.it (L.P.); 21 UOC di Neonatologia/TIN, Dipartimento Materno Infantile, Azienda Ospedaliera di Cosenza, 87100 Cosenza, Italy; g.scarpelli@aocs.it (G.S.); e.bonanno@aocs.it (E.B.); 22 UO di Pediatria/Nido, Materno Infantile, ASST Pavia, 27029 Vigevano, Italy; lidia_decembrino@asst-pavia.it; 23 SC Pediatria e DEA Pediatrico, AOU SS Antonio e Biagio e C. Arrigo, 15121 Alessandria, Italy; enrico.felici@ospedale.al.it (E.F.); camilla.selvatico@ospedale.al.it (C.S.); valentina.saracco@ospedale.al.it (V.S.); 24 UOC Pediatria e Assistenza Neonatale, Dipartimento Materno Infantile, Spoke Corigliano Rossano, Azienda Sanitaria Provinciale (ASP) di Cosenza, 87067 Rossano, Italy; f.morrone@aspcs.it; 25 Department of Health Promotion Sciences, Maternal and Infant Care, Internal Medicine and Excellence Specialties, University of Palermo, 90127 Palermo, Italy; claudio.costantino01@unipa.it; 26 UOC Neonatologia con Nido e TIN, PROMISE Department, Azienda Ospedaliera Universitaria Policlinico “Paolo Giaccone”, University of Palermo, 90127 Palermo, Italy; mario.giuffre@unipa.it

**Keywords:** respiratory syncytial virus, human metapneumovirus, respiratory virus, respiratory infections, monoclonal antibodies, lower respiratory tract infections

## Abstract

Nirsevimab is a long-acting monoclonal antibody designed to prevent infections due to respiratory syncytial virus (RSV). Here we report on a retrospective, multicenter study encompassing a total of 19 Italian neonatal and pediatric centers evaluating the epidemiology of lower respiratory tract infection (LRTI)-related hospitalizations in infants younger than 2 years during the first RSV season following the introduction of nirsevimab prophylaxis. A total of 401 hospitalizations were reported, with 40.4% being in children with previous prophylaxis with nirsevimab. Respiratory syncytial virus was the most frequently identified pathogen (47.5%), followed by rhinovirus/enterovirus (20.2%) and human metapneumovirus (hMPV; 6.9%). In multivariable analyses adjusted for age, sex, and month of diagnosis, prior nirsevimab immunization was associated with a significantly reduced likelihood of RSV-related hospitalization (adjusted odds ratio [aOR], 0.259; 95% CI, 0.157–0.427), corresponding to an estimated effectiveness of 74.1% (95% CI, 57.3–84.3). Conversely, nirsevimab-immunized infants showed increased odds of hospitalization due to hMPV (aOR, 2.490; 95% CI, 1.019–6.085) and rhinovirus/enterovirus (aOR, 2.573; 95% CI, 1.424–4.650). Lower respiratory tract infections associated with hMPV predominantly occurred outside the typical RSV season, being associated with moderate-to-severe clinical presentations. These findings confirm the real-world effectiveness of nirsevimab against RSV hospitalizations, also highlighting the need for the continued surveillance of non-RSV respiratory pathogens in the context of universal RSV immunoprophylaxis.

## 1. Introduction

Lower respiratory tract infections (LRTIs; i.e., infections affecting the airways below the level of the laryinx, including acute bronchitis, acute exacerbations of chronic bronchitis, bronchiolitis, pneumonia) [1] are a leading cause of morbidity in infants and children under two years of age, representing a major global health concern [2,3,4]. In this population, LRTIs account for a substantial proportion of hospital admissions, emergency presentations, and episodes requiring advanced respiratory support [1,2,3,4,5,6]. A wide range of pathogens contribute to LRTIs in the first two years of life [5,7,8]. The most common infectious agents are viruses, particularly respiratory viruses such as rhinovirus (RIV) and enterovirus (ENT), coronavirus (CoV), including SARS-CoV-2, adenovirus, pandemic and seasonal influenza virus A and B (IFV), and parainfluenza virus (PIV) [1,4,5]. Among the myriad of pathogens responsible of LRTIs in children, two closely related viruses belonging to the *Pneumoviridae* family are considered major causes of LRTI at global level: respiratory syncytial virus (RSV), and human metapneumovirus (hMPV) [9,10,11,12].

RSV and hMPV are small, enveloped, negative-sense, single-stranded RNA viruses with a genome whose length ranges between 13 kb (hMPV) [13] and approximately 15.2 kb (RSV) [14], sharing similarities in their structures, viral life cycles, and strategies for host interaction and immune evasion [13,14,15,16]. Both pathogens are strongly associated with bronchiolitis, viral pneumonia, and respiratory failure in all age groups, and had extensively co-circulated until the COVID-19 pandemic [9,17,18,19,20,21]. Despite advances in pediatric care, the clinical and healthcare burden associated with these infections remains considerable, with recurrent seasonal surges causing significant pressure on hospital systems, especially in temperate regions. Currently, the global burden of RSV is approximately around 3.6 million hospitalizations of infants and children per year, while hMPV would account for 5–10% of children hospitalizations due to LRTI [22], with high heterogeneity across age groups [12,13,16,23,24,25,26].

The preventive landscape for RSV has profoundly changed in recent years [27,28], as it now includes innovative options such a long-acting monoclonal antibodies (mAbs) for newborns and infants (i.e., nirsevimab and clesrovimab), one maternal vaccine for indirect protection of newborns in their first RSV season (i.e., RSVpreF) [29,30,31], and effective vaccines for adults and elderly (i.e., RSVpreF, commercial name ABRYSVO^®^, Pfizer Inc. New York, USA; RSVpreF3, commercial name AREXVY^®^, GlaxoSmithKline [GSK] PLC, Brentford, London, United Kingdom; and mRNA-1345, commercial name mResvia^®^, Moderna Biotech SL, Cambridge, Massachusetts, USA [32,33], and similar options will be reasonably made available for hMPV [34]. These interventions, and particularly the introduction of the long-acting mAb nirsevimab, did substantially reduce RSV-related hospitalizations in newborns and infants [35,36,37,38,39,40,41,42]. Administered as a single intramuscular dose, it provides season-long protection and is now recommended for nearly all infants entering their first RSV season in many countries [14,27,28,37,43]. Currently, the systematic implementation of nirsevimab has not been fully embedded within the Italian National Immunization Prevention Plan, leading to marked regional variability in its uptake nationwide [27,33,41,44,45].

While the real-world evidence on maternal vaccination and mAb quite consistently stresses the high effectiveness of both strategies in avoiding RSV-related LRTIs among recipient infants and children [27,29,30,31,40,41,46,47,48,49], their broader impact on the whole of LRTI epidemiology remains uncertain. As the selective pressure exerted on RSV by mAb and maternal vaccines may lead to a decline in RSV incidence, it is unclear whether it may alter the relative contribution of other pathogens, shift seasonal patterns, or influence viral interference and co-infection dynamics. Due to its evolutive relation and to the extensive similarities with RSV, hMPV may represent a suitable candidate for its eventual replacement [9,16], but the extent to which non-RSV viruses, including hMPV, may fill the ecological space vacated by declining RSV circulation is currently unknown.

A precise understanding of the epidemiology and clinical impact of respiratory viruses during the first seasons benefiting from RSV-preventive interventions and across different epidemiological contexts and delivery models is therefore essential for informing prevention strategies and optimizing patient management. Therefore, we designed a multicenter, retrospective, test-negative, case–control study including a total of 19 hospitals in various Italian Regions (Appendix A Table A1) reporting on subjects hospitalized because of LRTI during the winter season 2024–2025 (see STROBE Checklist as Supplementary File S1).

## 2. Materials and Methods

### 2.1. Data Source

**Figure 1:**
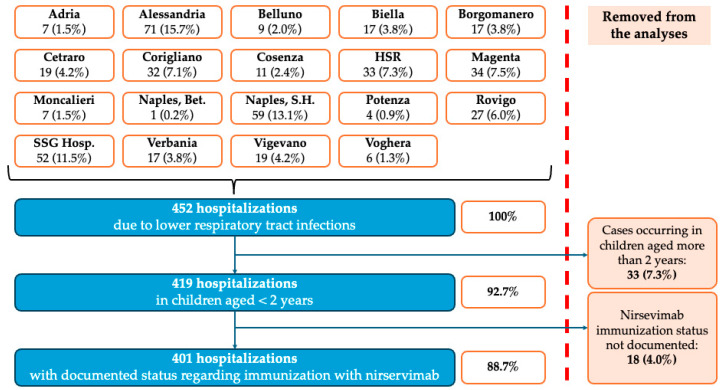
Flowchart of included cases by participating centers. Note: HSR = San Raffaele Hospital, Milan; Naples, Bet = Evengelic Betania Hospital, Naples; Naples S.H. = Santobono Pausilipon Hospital, Naples; SSG Hosp. = Sesto San Giovanni Hospital, Milan.

Health records from centers participating in the present multicenter study (Figure 1) were retrospectively analyzed in order to capture all hospitalizations due to LRTI occurring during the time period from 1 November 2024 to 30 April 2025 (i.e., the first RSV season benefiting from nationwide availability of nirsevimab). Inclusion criteria were the following: (1) being suitable for delivery of nirsevimab, i.e., age < 5 months by 1 November 2024; (2) age at hospitalization < 24 months; (3) clinical condition codified as J21.- according to the 10th revision of the International Classification of Diseases (ICD); (3) availability of laboratory work-up on respiratory specimens, including real-time polymerase chain reaction (RT-PCR) testing for the following respiratory pathogens: RSV, INF A, INF B, RIV/ENT, hMPV, adenovirus, bocavirus, non-pandemic CoV, SARS-CoV-2, PIV; and (4) status regarding immunization with nirsevimab (i.e., immunized vs. not immunized).

The following data were eventually retrieved: gender, age (in months) at hospitalization, length of hospital stay (days), need for respiratory support with either low- or high-flow nasal cannula, admission to pediatric intensive care unit (PICU), and eventual outcome (i.e., eventual discharge at home or LRTI-related death). No additional data were collected in order to preserve the anonymity of the identified cases.

### 2.2. Analysis of Main Data

Our study included infants aged 24 months or younger who were hospitalized with a diagnosis of LRTI between 1 November 2024 and 30 April 2025; as a working definition, cases were considered of interest for the present study if associated with one of the following International Classification of Diseases (ICD)-10 codes: J10.0; J11.0; J12, J13; J14; J15; J18; J20; J21; J22; J44; or J90). All eligible cases were tested for RSV, INF A, INF B, RIV/ENT, hMPV, adenovirus, bocavirus, non-pandemic CoV, SARS-CoV-2, and PIV using RT-PCR. The following exclusion criteria were conversely implemented: (a) cases occurring in children aged 24 months or older; (b) hospitalizations for LRTI without microbiological laboratory testing aimed at identifying the underlying respiratory pathogen; (c) cases of undocumented nirsevimab prophylaxis status; and (d) cases initially treated with palivizumab and subsequently switched to nirsevimab. For the analysis of nirsevimab immunization effectiveness (IE), all infants with any positive RSV test were considered RSV-positive cases, while controls were all subjects with a RT-PCR-confirmed negative status for RSV.

### 2.3. Ethical Considerations

All data were handled in accordance with the ethical principles outlined in the Declaration of Helsinki. The study protocol was reviewed and approved by the Ethics and Research Committees of each participating institution. All datasets were anonymized before analysis, and access to the database was restricted to authorized research personnel at each study center. Individual informed consent was not required, and a waiver of consent was granted by the local Ethics Committees.

### 2.4. Statistical Analysis

As a preliminary step, a descriptive analysis was performed. Continuous variables were reported as the mean ± standard deviation (SD), being preliminarily tested for normality by means of the K2 test, with significance level of α = 0.10 implemented in order to increase the test’s sensitivity to departures from normality. As a consequence, a K2 test *p*-value of <0.100 was considered the cut-off for considering a certain variable as not normally distributed variables, leading to its analysis by means of the Mann–Whitney or Kruskal–Wallis tests, while the correlation analysis with other variables required Spearman’s rank test. Conversely, variables associated with a K2 test *p*-value of ≥0.100 were considered normally distributed, and their correlation was assessed by means of Pearson’s correlation coefficient.

Levene’s test was then used to assess the equality of variances (i.e., homoscedasticity) of the data. Data exhibiting homoscedasticity and normality were compared between the groups by means of an independent-samples *t*-test. Heteroscedastic normal data were compared between the groups with the Welch test (unequal variance *t*-test). In cases of multiple comparisons, Analysis of Variance (ANOVA) with post hoc Dunnet’s test was performed.

Categorical variables were reported as percent values, including the detection rates (i.e., the number of cases of a certain pathogen over the total of included hospitalizations). Detection rates were calculated both on the whole of included cases and as a pooled analysis by means of a meta-analysis approach. A random-effect model (REM) was chosen over a fixed-effect one in order to cope with the presumptive heterogeneity epidemiology of respiratory viruses due to the multicenter design of the present study. Within a REM, the influence of categorical variables is assumed to vary across different levels of the variable. In other words, because random-effect models account for variability and differences across populations or study settings, this approach was deemed more suitable to address the true heterogeneity underlying the study findings [50,51]. The pooled detection rates for hMPV were then compared to those for RSV and RIV/ENT by calculation of the Risk Ratios (RR) and their corresponding 95% confidence intervals (95% CIs). Heterogeneity, i.e., the inconsistency of the effect between the included centers or the percentage of total variation across the included centers likely due to actual differences rather than chance [52], was quantified in percent values by means of I^2^ statistics. In accordance with current recommendations, heterogeneity was considered low for I^2^ values ≤ 25%, moderate for I^2^ values ≥ 26% and <50%, and substantial for I^2^ values ≥ 50% [52,53,54]. For each I^2^ estimate, 95% CIs were calculated and reported [52].

The distribution of categorical variables between cases of RSV, hMPV, and RIV/ENT were assessed by the chi squared test. Missing values for assessed variables were handled by means of listwise deletion, i.e., observations with incomplete data were excluded from the specific analysis in which the missingness occurred. In all of the aforementioned analyses, a *p* value < 0.05 was considered statistically significant. Similarly, the occurrence of virological diagnoses by having been immunized or not with nirsevimab was assessed by the chi-squared test and the calculation of corresponding odds ratios (ORs). All associations between a certain virological diagnosis and the previous delivery of nirsevimab with a *p* < 0.05 were then assessed by calculation of the multivariable adjusted OR (aOR) by a binary logistical regression model that included age, gender, and month of hospitalization as covariates.

Statistical analyses were performed using IBM SPSS Statistics 26.0 for Macintosh (IBM Corp. Armonk, NY, USA), GraphPad Prism Version 10.6.1 (799) (GraphPad Software LLC, San Diego, CA, USA), R (version 4.4.1) [55], and RStudio 2026.01.0 Build 392 (Posit Software PBC, Boston, MA, USA) using the packages fmsb (version 0.7.6), ggplot2 (version 4.0.1), ggpubr (version 0.6.2), and meta (version 8.2.1).

## 3. Results

### 3.1. Demographic Features of Sampled Population

During the winter season 2024–2025, a total of 452 hospitalizations due to LRTIs were reported from participating centers. Of them, 419 subjects were under two years old (92.7%); 401 of these had received nirservimab and were included in the data analysis (88.7% of the original population) (Figure 1).

**Figure 2:**
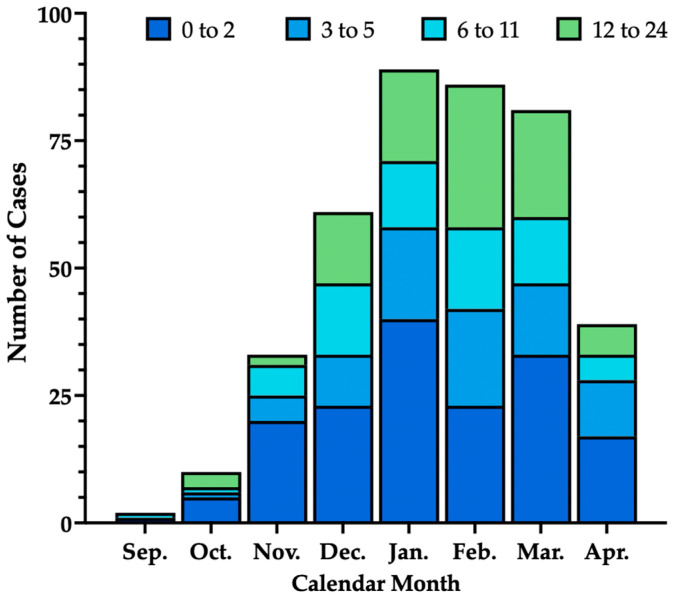
Seasonal trend of hospitalizations due to lower respiratory tract infections from the participating centers by age at admission (in months) (n = 401).

Overall, 228 were males (56.9%), with a mean age of 7.2 months ± 8.1. Most cases (79.1%) were reported in December–March, with a mean hospital stay of 5.2 days ± 2.8: of 401 sampled children, 162 (40.4%) were aged less than 3 months, with 78 (19.5%) aged 3–5 months, 69 (17.2%) aged 6–11 months, and 92 (22.9%) aged 12–24 months (Figure 2).

As shown in Figure 2, hospitalizations increased from September (0.5%) through the months of October (2.6%), November (8.4%), and December (15.0%), peaking during the month of January (22.4%), with a sustained high rate across the months of February (21.0%) and March (20.8%), then decreasing to 9.3% in the month of April.

No deaths were reported, but 35.4% of cases required O_2_ therapy, including high-flow nasal cannula (26.7%), with 23 cases (5.7%) requiring PICU admission.

### 3.2. Virological Diagnoses

Most LRTI cases were due to a single pathogen (86.8%), while 13.2% were associated with two (12.0%) or three (1.2%) pathogens (Table 1). The most frequently documented viral agent was RSV (47.4%), followed by rhinovirus/enterovirus (20.2%), hMPV (7.2%), and seasonal influenza (7.2%, including INF A, 5.0%, and INF B, 2.2%), adenovirus (3.5%), non-pandemic human coronaviruses (3.2%), parainfluenza virus (1.2%), bocavirus (0.7%), and SARS-CoV-2 (0.5%). Only three cases were associated with bacterial pathogens, i.e., 2 episodes associated with *Mycoplasma pnemoniae* (0.5%) and one case associated with *Streptococcus pneumoniae* (0.2%).

**Figure 3:**
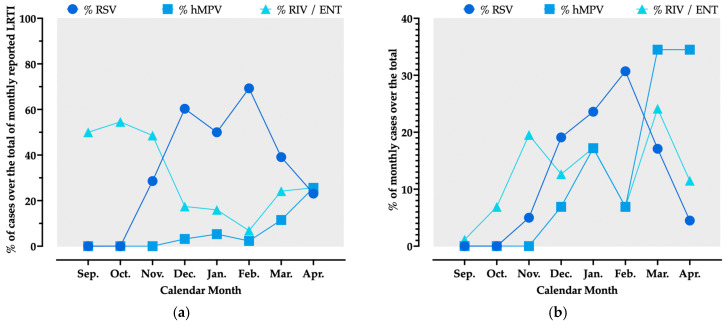
Trend of hospitalizations due to human metapneumovirus (hMPV), respiratory syncytial virus (RSV), and rhino- and enterovirus (RIV/ENT), reported as percent values over the total of monthly lower respiratory tract infection cases (**a**) and over total of cases per pathogen (**b**).

The seasonal trend of RSV, hMPV and RIV/ENT is shown in Figure 3. Briefly, RIV/ENT exhibited relatively low detection rates during the months of September and October (1.2% and 6.9% of total cases documented during the assessed timeframe). However, as no cases of RSV and hMPV were reported, RIV/ENT contributed to around half of all isolates (50.0% and 54.6% for September and October, respectively). The proportion of RSV cases over the whole of LRTI increased through the months of November (28.6%), December (60.3%), and January (50.0%), peaking to 69.3% in February and then decreasing to 39.1% in March and 23.1% in April (Figure 3a). Conversely, during the winter months of December, January, and February, the proportion of RIV/ENT over the monthly isolates remained relatively low (17.5%, 16.0%, and 6.8%, respectively), with a subsequent surge during the months of March (24.1%) and April (25.6%). A similar trend was identified for hMPV, whose proportion over the total of monthly detected pathogens ranged from 3.2% in December, 5.3% in January, 2.3% in February, increasing to 11.5% in March and peaking at 25.6% in April.

Comparing the proportions of RSV and hMPV cases over the whole of hospitalizations due to these pathogens, around three quarters of RSV cases occurred across the months December (19.1%), January (23.6%), and February (30.7%), while a similar proportion of hMPV cases occurred from February (6.9%) to March (34.5%) and eventually April (34.5%). Conversely, around 80% of RIV/ENT cases clustered between November and March (Figure 3b).

**Figure 4:**
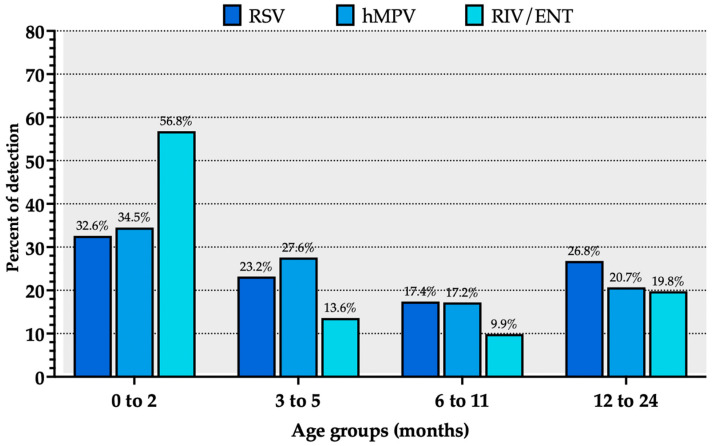
Trend of hospitalizations due to human metapneumovirus (hMPV), respiratory syncytial virus (RSV), and rhino- and enterovirus (RIV/ENT) according to age groups in months, reported as percent values over all of the LRTI cases.

Among the sampled children, more than half of cases of RIV/ENT (56.8%) occurred in the 0–2 months age group compared to 32.6% of RSV cases and 34.5% of hMPV (Figure 4). The proportion of RIV/ENT cases decreased in the older age groups following a U-shaped curve, ranging between 13.6% in the 3–5 months age group, 9.9% among children aged 6–11 months, and 19.8% among children aged 12–24 months. A similar trend was identified for RSV (23.2%, 17.4%, and 26.8% of cases for age groups 3–5 months, 6–11 months, and 12–24 months, respectively) and hMPV cases (34.5%, 27.6%, 17.2%, and 19.8% for age groups 3–5 months, 6–11 months, and 12–24 months, respectively).

**Figure 5:**
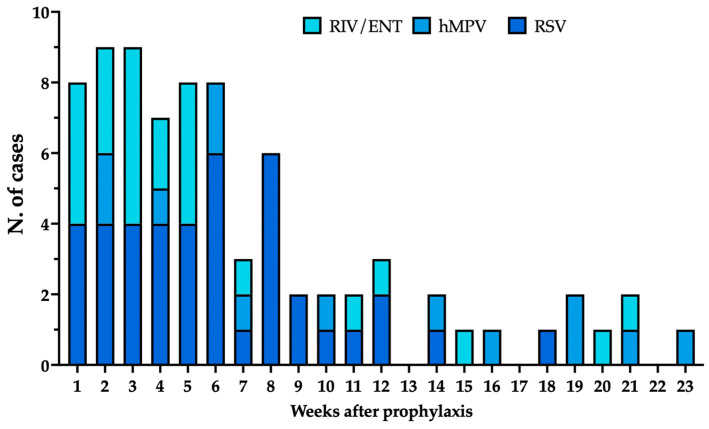
Trend of hospitalizations due to human metapneumovirus (hMPV), respiratory syncytial virus (RSV), and rhino- and enterovirus (RIV/ENT) according to the time (weeks) elapsed since the prophylaxis with nirsevimab.

Focusing on cases which benefited from prophylaxis with nirsevimab, hospitalizations occurred within an average timespan of 48.1 days ± 38.7, i.e., 42.0 days ± 26.7 for RSV, 80.5 days ± 52.2 for hMPV, and 40.5 days ± 40.2 for RIV/ENT. As shown in Figure 5, most cases of breakthrough for RSV occurred during the first weeks after prophylaxis, with a subsequent decrease (Spearman’s rho −0.862, 95% CI from −0.942 to 0.691, *p* < 0.001), while no correlation was found for hMPV (Spearman’s rho = −0.028, 95% CI from −0.445 to 0.400, *p* = 0.899). Interestingly, a negative correlation was also identified between prophylaxis with nirsevimab and hospitalization due to RIV/ENT-associated LRTI (Spearman rho = −0.568, 95% CI from −0.799 to −0.192, *p* = 0.005).

### 3.3. Meta-Analysis of Detection Rates

**Figure A1:**
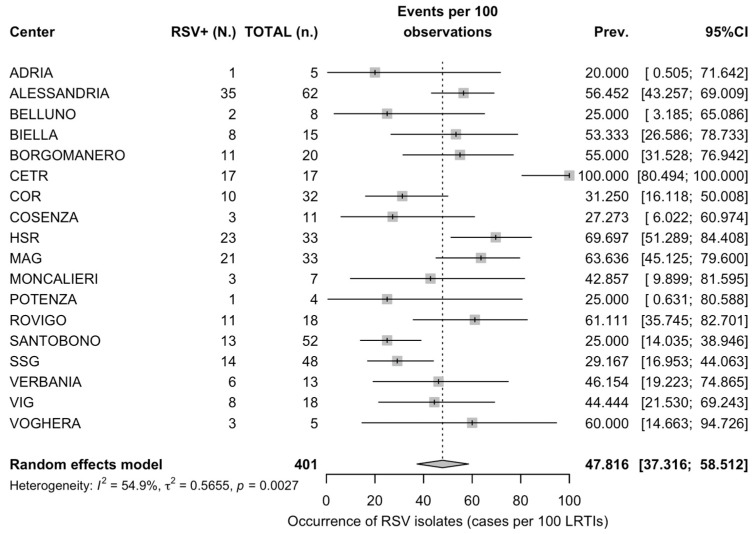
Detection rates for respiratory syncytial virus (RSV) among hospitalizations due to lower respiratory tract infections (LRTIs) from participating centers. A pooled estimate of 47.8 cases per 100 hospitalizations (95% confidence interval [95% CI] from 37.3 to 58.5) was calculated, with moderate to substantial heterogeneity (I^2^ 54.9%, 95% CI from 23.3 to 73.5).

**Figure A2:**
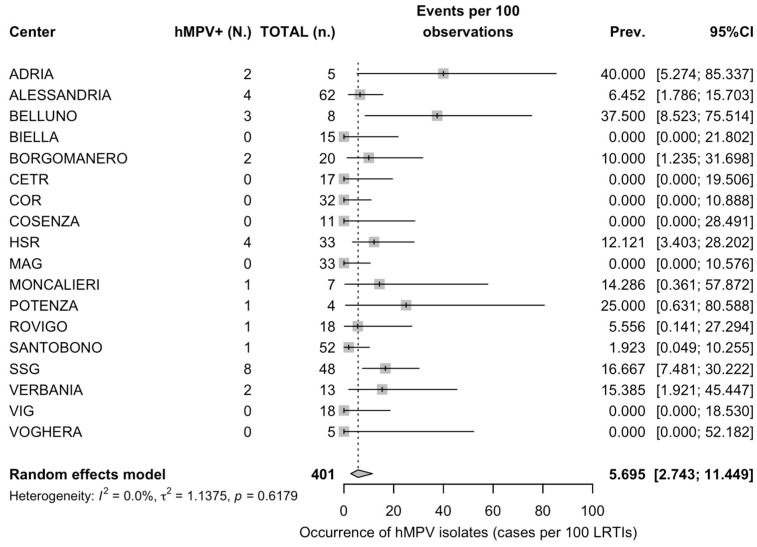
Detection rates for human metapneumovirus (hMPV) among hospitalizations due to lower respiratory tract infections (LRTIs) from participating centers. A pooled estimate of 5.7 cases per 100 hospitalizations (95% confidence interval [95% CI] from 2.7 to 11.4) was calculated, with low to moderate heterogeneity (I^2^ 0.0%, 95% CI from 0.0 to 50.0).

**Figure A3:**
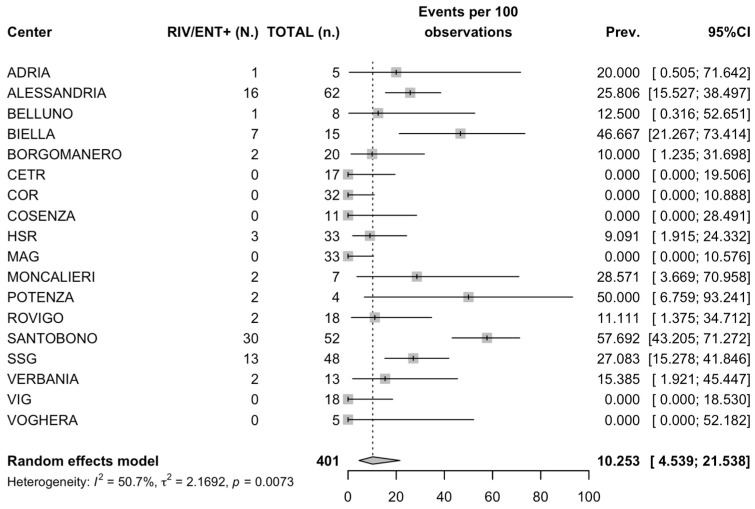
Detection rates for rhino- and enterovirus (RIV/ENT) among hospitalizations due to lower respiratory tract infections (LRTIs) from participating centers. A pooled estimate of 10.3 cases per 100 hospitalizations (95% confidence interval [95% CI] from 4.5 to 21.5) was calculated, with moderate to substantial heterogeneity (I^2^ 50.7%, 95% CI from 15.2 to 71.3).

A pooled detection rate for RSV was estimated into 47.8 per 100 hospitalizations (95% CI from 37.3 to 58.5) compared to 5.7 per 100 hospitalizations (95% CI from 2.7 to 11.4) for hMPV and 10.3 per 100 hospitalizations (95% CI from 4.5 to 21.5) for RIV/ENT (Appendix A Figure A1, Figure A2 and Figure A3). In fact, all estimates were affected by moderate-to-substantial heterogeneity (i.e., I^2^ 54.9%, 95% CI from 23.3 to 73.5 for RSV, I^2^ 0.0%, 95% CI from 0.0 to 50.0 for hMPV, and I^2^ 50.7%, 95% CI from 15.2 to 71.3 for RIV/ENT).

**Figure A4:**
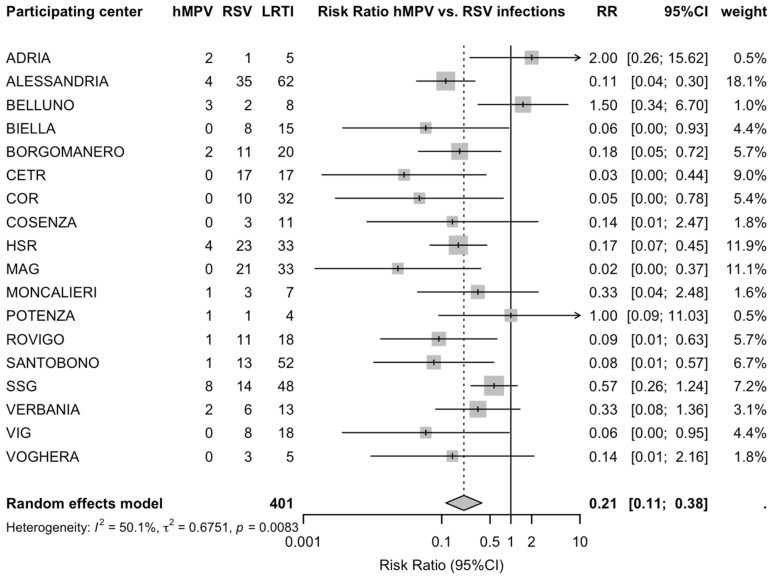
Comparison of detection rates for human metapneumovirus (hMPV) vs. respiratory syncytial virus (RSV) among hospitalizations due to lower respiratory tract infections (LRTIs) from participating centers. A pooled estimate of RR 0.209 (95% confidence interval [95% CI] from 0.115 to 0.381) was calculated, with moderate to substantial heterogeneity (I^2^ 50.1%, 95% CI from 14.1 to 71.0).

**Figure A5:**
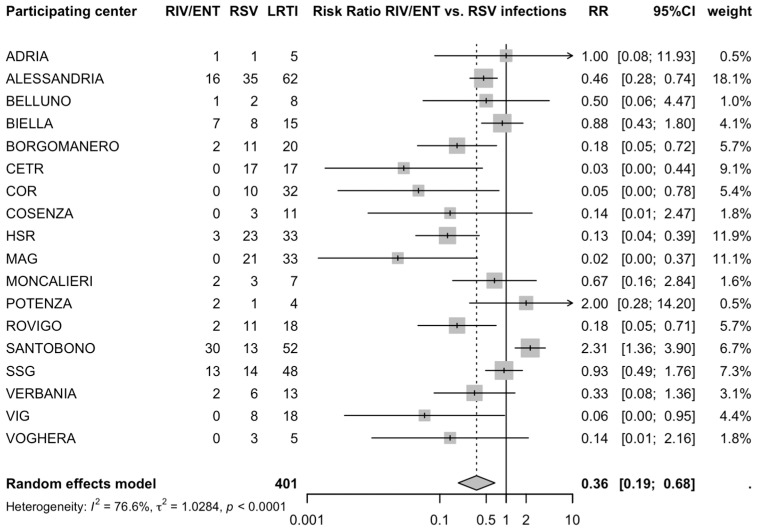
Comparison of detection rates for rhino- and enterovirus (RIV/ENT) vs. respiratory syncytial virus (RSV) among hospitalizations due to lower respiratory tract infections (LRTIs) from participating centers. A pooled estimate of RR 0.358 (95% confidence interval [95% CI] from 0.189 to 0.677) was calculated, with substantial heterogeneity (I^2^ 76.6%, 95% CI from 63.2 to 85.1).

The pooled likelihood for detection of either hMPV or RIV/ENT among hospitalized LRTI was significantly lower than that for RSV, with an RR of 0.209 (95% CI from 0.114 to 0.381) for hMPV vs. RSV (Appendix A Figure A4) and an RR of 0.358 (95% CI from 0.189 to 0.677) for RIV/ENT vs. RSV (Appendix A Figure A5). Both analyses were affected by substantial heterogeneity, with a I^2^ 50.1% estimate for hMPV vs. RSV detections (95% CI from 14.1 to 71.0) and I^2^ 76.6% for RIV/ENT vs. RSV detections (95% CI from 63.2 to 85.1).

### 3.4. Analysis of Main Risk Factors

As shown in Table 2, the proportion of male cases was slightly greater among RSV than among hMPV cases (58.9% vs. 51.7%), but the difference was not significant (*p* = 0.463). Conversely, the proportion of males was significantly lower than that for females for RIV/ENT (42.0% vs. 58.0%, *p* = 0.011). Similarly, cases with a diagnosis of RSV infection were not significantly older than subjects hospitalized because of LRTI associated with hMPV infections (8.2 months ± 9.2 vs. 7.0 months ± 6.2; *p* = 0.369) and RIV/ENT (6.6 months ± 10.1, *p* = 0.220).

**Figure 6:**
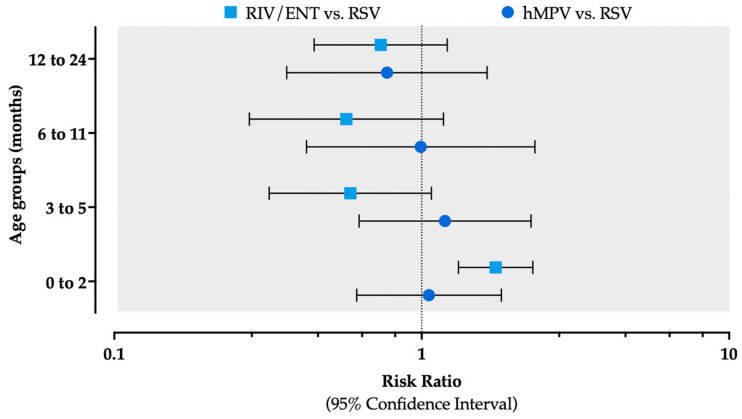
Trend of hospitalizations due to human metapneumovirus (hMPV), respiratory syncytial virus (RSV), and rhino- and enterovirus (RIV/ENT), reported as percent values over all of the LRTI cases by age group.

Taking the detection rate for RSV as the reference category, an increased risk of hospitalization due to RIV/ENT was identified among children aged 0–2 months (RR 1.740, 95% CI from 1.317 to 2.300), while no substantial differences were identified across the remaining age groups (Figure 6, Appendix A Table A1).

With respect to the month of diagnosis, a significantly greater proportion of cases occurred between April and November for both hMPV (34.5%) and RIV/ENT (39.5%) compared to the LRTIs associated with RSV infection (10.0%; *p* < 0.001 for both comparisons): assuming the detection rate for RSV as the reference category, an increased risk for both hMPV and RIV/ENT was therefore documented for the timeframe from April to December (RR 3.448, 95% CI from 1.785 to 6.662, and RR 3.951, 95% CI from 2.385 to 6.543, respectively), which was mirrored by a similarly decreased risk during the timeframe from December to March (RR 0.728, 95% CI from 0.557 to 0.952, and RR 0.672, 95% CI from 0.560 to 0.807 for hMPV and RIV/ENT, respectively; Appendix A Table A1).

When dealing with outcome variables such as the length of hospital stay (6.4 ± 2.3 vs. 6.5 ± 2.3 for hMPV and RSV, respectively), as well as the proportion of cases requiring PICU admissions (3.4% vs. 5.0%), no significant differences were identified between hMPV and RSV (*p* > 0.05). Conversely, RIV/ENT cases were characterized by a significantly shorter hospital stay (5.3 ± 2.9, *p* = 0.001) despite a greater proportion of PICU admission (12.3%, *p* = 0.031). The need for O_2_ therapy (51.7% for RSV vs. 43.2% for hMPV and 34.6% for RIV/ENT), including high-flow nasal cannula (41.4% for RSV vs. 30.7% for hMPV and 39.6% for RIV/ENT), were not differently distributed (*p* > 0.05). Finally, while no significant differences were reported regarding the proportion of cases associated with multiple virological diagnoses for hMPV (20.6%) compared to RSV cases (11.6%; *p* = 0.382), RIV/ENT cases were more frequently characterized as co-infections of two or more pathogens (40.7%, *p* < 0.001).

While more than half of hMPV (55.2%) and RIV/ENT cases (53.1%) were reportedly immunized by means of nirsevimab, the proportion of RSV cases was significantly lower (27.4%; *p* = 0.003 and *p* < 0.001, respectively). As shown in Table 3, by applying a case-negative approach, nirsevimab immunization was associated with decreased odds of LRTI-related hospitalization (OR 0.346, 95% CI from 0.227 to 0.527; *p* < 0.001), for a crude estimate on immunization effectiveness of 65.4% (95% CI from 47.3 to 77.3%), while a non-significant rise in the likelihood for hMPV-related hospitalizations was noted among immunized children (OR 1.905, 95% CI from 0.890 to 4.077), and a significant increase in hospitalizations due to RIV/ENT was identified (OR 1.911, 95% CI from 1.169 to 3.125).

In a multivariable model including age at hospitalization, gender, and month of diagnosis as covariates, significantly decreased odds for hospitalizations due to RSV were associated with previous immunization with nirsevimab (aOR 0.259, 95% CI from 0.157 to 0.427), with a subsequent estimate for immunization effectiveness of 74.1% (95% CI from 57.3 to 84.3).

On the contrary, newborns immunized with nirsevimab resulted in a significantly increased odds of hospitalizations for two respiratory viruses such as hMPV (aOR 2.490, 95% CI from 1.019 to 6.085) and RIV/ENT (aOR 2.573, 95% CI from 1.424 to 4.650), but not for other ones analyzed in the RT-PCR Panel (INF A and B, Adenovirus, Bocavirus, PIV, Non Pandemic CoV).

## 4. Discussion

### 4.1. Summary of Main Findings

We report on a retrospective, multicenter study from 19 Italian neonatal and pediatric centers. Overall, we documented a total of 401 hospitalizations due to LRTI episodes in infants (age < 2 years) with a documented status regarding the previous uptake of nirsevimab: of them, 40.4% were actually immunized in accord with the official recommendations in effect at the time [28,33]. The most frequently reported pathogen was represented by RSV (47.5%) followed by RIV/ENT (20.2%), while hMPV ranked as the third most frequent viral agent with 6.9% of cases. Notably, 86.8% of cases were represented by single virological pathogen diagnoses, while only 3% were associated with bacterial pathogens.

Compared to RSV cases, hMPV cases exhibited no significant differences for gender, age at diagnosis, or geographic location. On the contrary, LRTIs caused by hMPV were more likely to occur in the timeframe April–December (aOR 7.052, 95% CI from 2.436 to 20.415) and in infants previously immunization with nirsevimab (aOR 2.490 from 1.019 to 6.085).

Only 35.4% of hospitalized cases required any supplemental O_2_: available clinical data suggest a generally moderate-to-high severity of hMPV LRTIs, as more than half of hospitalized infants required any oxygen supplementation (51.7%), compared to 41.6% of RSV and 34.6% of RIV/ENT. Interestingly, only one case of hMPV-associated LRTI required the eventual admission to a NICU/PICU (3.4%), compared to 5.3% of RSV and 12.3% of RIV/ENT.

### 4.2. Generalizability of Main Results

Respiratory viruses are common pathogens, extensively associated with high incidence and hospitalization rates [56,57,58]. Both in pre- and post-pandemic settings, RSV was always documented in Italy as the most commonly reported pathogen in pediatric hospitalizations due to respiratory viruses, while the ratio of hMPV to RSV cases was very heterogenous, ranging from 1:1.94 to 1:4.23 and even 1:7.42 [59,60,61]. Also, in our study, the ratio of RSV:hMPV was estimated as 6.82 (95% CI from 2.65 to 11.07), and the actual ratio of cases ranged from 0.67 to 18.00. A male predominance was noted for RSV hospitalized cases. These results are substantially consistent with the findings from previous studies [62,63,64,65,66,67,68], suggesting a greater susceptibility amongst males for more severe disease and hospitalization due to RSV infections, whether using case–control, cohort, or pooled retrospective analyses. The absence of gender as a finding for hMPV is also noteworthy [10,11,69,70], and the difference in these observations deserves further study, specifically their interactions with respiratory epithelium, as well as with innate immune mechanisms. Notably, RIV/ENT were associated with shorter hospital stay, despite greater PICU admission, but these results are consistent with more recent studies underscoring the contribution of RIV to severe disease than previously assumed [71,72,73,74,75].

Within our multicenter study, the epidemiology of respiratory pathogens was highly affected by the geographic distribution of participating centers; a certain degree of heterogeneity was, to some extent, predictable, as Italy is characterized by marked climatic diversity through a North–South trend, with a resulting diachronous RSV seasonality [76,77]. These climatic differences translate into substantial regional variability in the onset of the winter season, with an earlier start and longer duration in the northern regions and a later onset with a shorter duration in the southern and insular areas [76,77,78]. Therefore, our study integrated some regional reports that collectively reported on the occurrence of RSV and hMPV in Italy during the first seasons that benefited from nirservimab preventive option [45,46,79,80,81]. In this regard, three main outcomes of our study could be of interest from both a national and international point of view.

First, our results are in line with previous estimates of the immunization efficacy of nirsevimab against hospitalizations due to RSV. In a previous preliminary report on 17 out of 19 participating centers [41], we estimated an immunization effectiveness of 65.5% (95% CI from 43.0 to 79.2) compared to the crude estimate of 65.4% (95% CI from 47.3 to 77.3%) and multivariable estimate of 74.1% (95% CI from 57.3 to 84.3) from the present report. These findings are therefore broadly consistent with the available Italian reports summarized in Appendix A Table A2, hinting to a crude immunization effectiveness ranging from 51.11% (95% CI from −40.70 to 83.05) [81] to 77.18% (95% from 49.60 to 88.86) [40]. In other words, as highly expected due to previously available evidence, the real-world effectiveness of nirsevimab was also proven from a nation-wide point of view [14,27,33]. Nonetheless, the comparison with available data, and particularly with earlier reports [81,82], also suggests that the expected effectiveness of nirsevimab in reducing hospitalizations due to LRTI can only be achieved by guaranteeing the clear and consistent availability of this preventative option [44,82]. Unfortunately, the uneven implementation of nirsevimab prophylaxis during the first RSV season in 2024–2025 did result in an uneven effectiveness [44], as captured by some previous reports [81,82].

**Figure A6:**
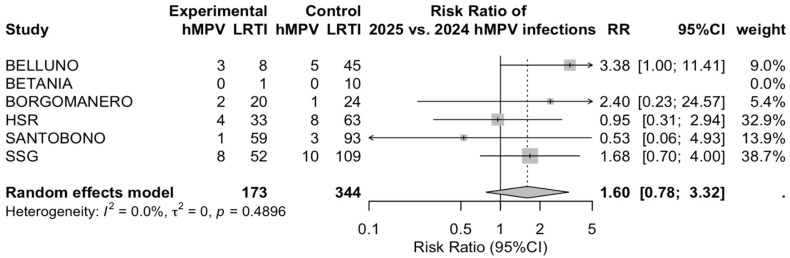
Comparison of detection rates for human metapneumovirus (hMPV) among hospitalizations due to lower respiratory tract infections (LRTIs) from participating centers in 2023–2024 vs. 2024–2025. A pooled estimate of RR 1.601 (95% confidence interval [95% CI] 0.777; 3.316) was calculated, with a seemingly low heterogeneity (I^2^ 0.0) not confirmed by analysis of 95% CI (95% CI 0.0 to 79.2).

Second, at least during the first complete RSV season benefiting from nirsevimab immunization, we did not find the substantial replacement of RSV from other pathogens and hMPV. In fact, not only was the pooled occurrence of reported infection was comparable with previous studies [59,60,61], but when data from participating centers on the winter season 2024–2025 were made available (Appendix A Figure A6), hospitalization rates were actually highly comparable (RR 1.601, 95% CI from 0.777 to 3.316), although affected by substantial heterogeneity (I^2^ 0.0, but 95% CI ranging from 0.0 to 79.2). On the other hand, in multivariable analysis, we found that previous prophylaxis with nirsevimab resulted in a paradoxically increased likelihood for LRTI hospitalizations due to hMPV (aOR 2.490, 95% CI 1.019; 6.085). Although seemingly conflicting, these results could be reconciliated by taking into account the following potential issues. On the one hand, due to the retrospective design of our study and the limited amount of personal data we were able to retrieve from institutional databases, we cannot rule out that personal, the individual risk factors (including social deprivation) and characteristics of the assessed RSV season in the parent Italian regions may have led to an increased occurrence of cases [83,84]. On the other hand, in the context of an Italian model of a universal healthcare system, the reduced occurrence of severe cases due to RSV following the implementation of nirsevimab prophylaxis could paradoxically guarantee the increased availability of medical options, sustaining hospital admissions because of other pathogens able to cause LRTI [83,84,85].

**Figure A7:**
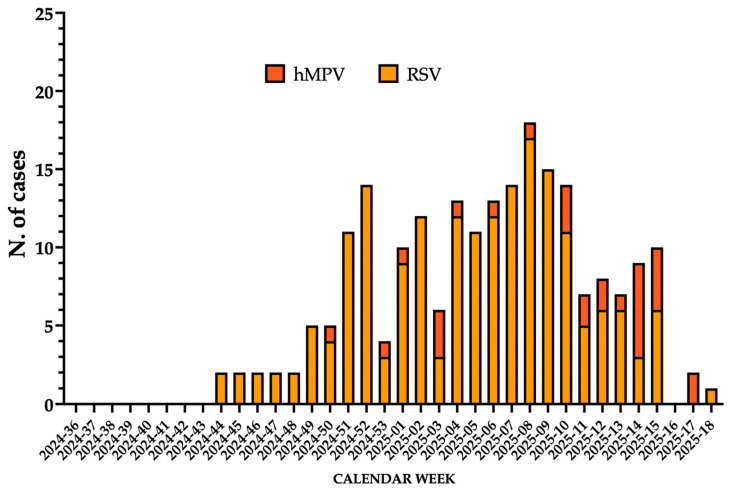
Seasonality of respiratory syncytial virus (RSV) and human metapneumovirus (hMPV) infections.

Third, our results are consistent with data suggesting that hMPV follows a similar but distinctive seasonal trend, whose peak would occur during the winter season, later than that for the RSV seasonal outbreak (Appendix A Figure A7) [17,22,86,87]. Usually, the hMPV peak has been reported from 4 to 8 weeks later than that of RSV, and again our results are substantially in line with the available evidence. As current research has led to the development of several potential mAbs targeting hMPV [88,89], the introduction of a prophylactic strategy against this pathogen will need to account for this discrepancy and for the need to integrate such approaches with other preventative strategies directed against RSV.

### 4.3. Implications for Medical Practice and Future Iterations

Our study suggests that, despite the actual effectiveness of nirsevimab in reducing hospitalizations due to RSV-related LRTI [27,37,43] with consequent cost savings [39,90], without a mass-immunization campaign that is able to reach all suitable children [91,92], RSV is likely to remain a substantial cause of consumption of healthcare resources in the following years [44]. Even though our data and available early reports do not suggest any shifts in viral pathogens causing LRTIs under conditions of universal prophylaxis against RSV with nirsevimab, the lessons learned with other respiratory pathogens such as pneumococcus [93,94,95], but also with viral pathogens such as seasonal influenza viruses [96,97,98] and SARS-CoV-2 [99,100,101], collectively stress the need for the early detection of viral replacements and/or antibody escape. More precisely, detecting as early as possible the surge of respiratory viruses historically less frequently reported than RSV will be the key for comprehensively reinforcing the strategies to prevent bronchiolitis—and not only RSV-associated bronchiolitis [57,102,103,104]—ultimately leading to an appropriate usage of healthcare resources [33,41,44].

### 4.4. Limits

Despite its potential significance, our study is affected by limitations. First and foremost, our study reports on a single RSV season, i.e., 2024–2025. Even though we embraced the first Italian RSV season actually benefiting from nirsevimab prevention [40,44,105], the seasonality of respiratory pathogens and their heterogenous occurrence over the years [80,106] collectively stress the need for a very cautious approach when dealing with hospitalization rates.

Second, although our study encompasses a total of 19 centers across the whole of the Italian peninsula, with a further participating center from the major island of Sicily, their characteristics were heterogenous, particularly with respect to catchment population, inpatient bed capacity, and the availability of therapeutic options for the inpatients. In other words, we cannot rule out that some participating centers may have reported inflated hospitalization rates for less common pathogens and/or more severe clinical features, impairing the reliability of our results. In effect, meta-analysis of hospitalization rates stressed the high heterogeneity of the available data, particularly when dealing with 95% CI of I^2^ statistics [53].

Third, respiratory infections are quite common in the general and pediatric populations [63,64,107], and the relatively small sample size could therefore be criticized. In this regard, it should be stressed that we included all incident hospitalizations from participating centers. Even though our estimates are therefore a direct representation of the actual seasonal epidemiology of respiratory pathogens, the relatively small sample size from each setting underscores the need for future studies to be conducted over multiple seasons in their setting. Nonetheless, by assuming an estimated point prevalence for RSV of 37.8% over the whole of acute respiratory infections [108], a total of 361 episodes of acute respiratory infections would be required for achieving a significance level of α = 0.05 [109], thereby indicating that our sample size is consistent with the requirements of our study outlines.

Fourth, our study implemented a retrospective design from local health records. Due to the requirements from competent Ethical Review Boards, only general data could be retrieved. Alongside main microbiological diagnoses and previous prophylaxis with nirsevimab, it only encompassed age at admission, gender, date of birth, PICU admission (when appropriate), and need for O_2_ treatment. On the other hand, personal information such as the status of preterm, weight at birth, and mostly any comorbidities were not available. As children and infants affected by respiratory and cardiovascular comorbidities are considered to be at high risk for severe complications due to LRTI, we cannot rule out the oversampling of “watch and wait” cases, i.e., subjects with mild symptoms benefiting from a precautionary hospitalization because of the underlying conditions. Still, because of the comprehensive approach we implemented—i.e., including all LRTI cases in the pooled sample the whole of LRTI cases—our study did likely retain the representativeness of the targeted population (i.e., children < 2 years of age) from the Italian regions with participating centers.

Finally, the source data did not provide a potential key issue, i.e., whether reported RSV infections, particularly breakthrough infections, are associated or not with the emergence of new variants, possibly exhibiting some degree of resistance to nirsevimab [110,111]. In fact, this is a critical issue that needs to be answered due to the unprecedented evolutionary pressure that nirservimab, maternal vaccination, and even vaccination of older adults are collectively exerting on RSV. Even though available studies suggest no evidence of nirsevimab escape mutations, the selection and spread of escape variants are a potential concern and need to be thoughtfully monitored [39,103,104,110,111].

## 5. Conclusions

In conclusion, our study provides nationwide evidence regarding possible modifications in the incidence rates of viruses other than RSV as causative agents of LRTIs needing hospitalization in a scenario of universal RSV passive immunization at birth with an anti-RSV mAb such as nirsevimab. Our data do not indicate a replacement of RSV by other pathogens, particularly hMPV; however, further studies are warranted to monitor the emergence of RSV variants resistant to nirsevimab and will require a longer observation time, including more than one or even two seasons. The precise identification and quantification of the different dynamics of infantile respiratory viruses causing LRTIs is urgently needed in order to tailor and optimize the preventative RSV strategies and to empower hospital pediatric services to manage the changing landscape of such viruses in young infants and children. We believe that our study moves in that direction, and we are looking forward to following up with similar epidemiological tracking during the ongoing second season of universal monoclonal antibody administration in Italy.

## Figures and Tables

**Table 1:** The main characteristics of 401 hospitalizations of children and infants (age < 2 years) due to lower respiratory tract infections included in the present multicenter study (Note: SD = standard deviation; PICU = pediatric intensive care unit).

Variable	Total (N/401, %) *
Gender	
Male	228, 56.9%
Female	173, 43.1%
Age (months ± SD)	7.2 ± 8.1
RSV season	
In season	221, 55.1%
Out of Season	180, 44.9%
Month of diagnosis	
April–November	84, 20.9%
December–March	317, 79.1%
Passively immunized with Nirservimab	162, 40.4%
Timespan from prophylaxis with Nirsevimab and hospitalization (days ± SD)	48.1 ± 38.7
Length of stay (days ± SD)	5.2 ± 2.8
O_2_ therapy	142, 35.4%
Of them, High-Flow nasal cannula	107, 26.7%
PICU admission	23, 5.7%
Virological diagnosis	
Single pathogen	348, 86.8%
Two pathogens	48, 12.0%
Three pathogens	5, 1.2%
Respiratory Syncytial Virus	190, 47.4%
Rhinovirus/Enterovirus	81, 20.2%
Human Metapneumovirus	29, 7.2%
Seasonal Influenza A	20, 5.0%
Seasonal Influenza B	9, 2.2%
Adenovirus	14, 3.5%
Human Coronavirus	13, 3.2%
Parainfluenza Virus	5, 1.2%
Bocavirus	3, 0.7%
SARS-CoV-2	2, 0.5%
Mycoplasma pneumoniae	2, 0.5%
Streptococcus pneumoniae	1, 0.2%

* *p* value calculated by means of chi squared test for categorical variables and Student’s *t* test for continuous variables.

**Table 2:** The main characteristics of hospitalizations due to human Respiratory Syncytial Virus (RSV), rhino- and enterovirus (RIV/ENT), and human Metapneumovirus (hMPV) in the children and infants (age < 2 years) included in the present multicenter study (Note: SD = standard deviation; PICU = pediatric intensive care unit).

Variable	RSV (N/190, %)	hMPV (N/29, %)	*p* Value *	RIV/ENT (N/81, %)	*p* Value *
Gender			0.463		0.011
Male	112, 58.9%	15, 51.7%		34, 42.0%	
Female	78, 41.1%	14, 48.3%		47, 58.0%	
Age (months ± SD)	8.2 ± 9.2	7.0 ± 6.2	0.369	6.6 ± 10.1	0.220
RSV season			0.130		0.160
In season	93, 48.9%	18, 64.3%		49, 60.5%	
Out of Season	97, 51.1%	10, 35.7%		32, 39.5%	
Month of diagnosis			<0.001		<0.001
April–November	19, 10.0%	10, 34.5%		32, 39.5%	
December–March	171, 90.0%	19, 65.5%		49, 60.5%	
Immunized with Nirservimab	52, 27.4%	16, 55.2%	0.003	43, 53.1%	<0.001
Length of stay (days ± SD)	6.5 ± 2.3	6.4 ± 2.3	0.827	5.3 ± 2.9	0.001
O_2_ therapy	79, 41.6%	15, 51.7%	0.304	28, 34.6%	0.182
Of them, High-Flow nasal cannula	57, 30.0%	12, 41.4%	0.219	24, 29.6%	0.866
PICU admission	10, 5.3%	1, 3.4%	0.677	10, 12.3%	0.031
Virological diagnosis **			0.382		<0.001
Single pathogen	168, 88.4%	23, 79.3%		48, 59.3%	
Two pathogens	19, 10.0%	5, 17.2%		30, 37.0%	
Three pathogens	3, 1.6%	1, 3.4%		3, 3.7%	

* *p* value calculated by chi squared test for categorical variables and Analysis of Variance (ANOVA) for continuous variables. RSV was considered as the reference category for all comparisons. ** due to rounding approximations, percentage totals may not sum exactly 100%.

**Table 3:** Association of immunization status with nirsevimab with hospitalizations in children and infants (age < 2 years) from participating centers due to lower respiratory tract infections. Adjusted Odds Ratios were calculated by means of logistic regression analysis; the model included age, gender, month of hospitalization as covariates. Note: RSV = human Respiratory Syncytial Virus; RIV/ENT = rhino- and enterovirus; hMPV = human Metapneumovirus; INF = seasonal influenza virus; CoV = coronavirus; PIV = parainflunzavirus.

Pathogen	Isolates (N/401, %)	Immunization with Nirservimab		Odds Ratio (95% Confidence Interval)	Adjusted Odds Ratio (95% Confidence Interval)
		Yes (n./N, %)	No (n./N)		
RSV	190, 47.4%	52, 26.1%	138, 73.9%	0.346 (0.227; 0.527)	0.259 (0.157; 0.427)
hMPV	29, 7.2%	16, 55.2%	13, 44.8%	1.905 (0.890; 4.077)	2.490 (1.019; 6.085)
INF A	20, 5.0%	12, 60.0%	8, 40.0%	2.310 (0.923; 5.784)	2.698 (0.875; 8.323)
INF B	9, 2.2%	6, 66.7%	3, 33.3%	3.026 (0.746; 12.277)	1.396 (0.291; 6.712)
Adenovirus	14, 3.5%	5, 35.7%	9, 64.3%	0.814 (0.268; 2.474)	3.577 (0.774; 16.526)
Bocavirus	3, 0.8%	0, -	3, 100%	-	-
PIV	5, 1.3%	3, 60.0%	2, 40.0%	2.236 (0.369; 13.532)	2.048 (0.261; 16.060)
Rhino/Enterovirus	81, 20.2%	43, 53.1%	38, 46.9%	1.911 (1.169; 3.125)	2.573 (1.424; 4.650)
Non pandemic CoV	13, 3.2%	6, 46.2%	7, 53.9%	1.275 (0.420; 3.865)	1.304 (0.367; 4.632)
SARS-CoV-2	2, 0.5%	1, 50.0%	1, 50.0%	1.478 (0.092; 23.805)	-
Coinfections RSV	17, 4.2%	5, 29.4%	12, 70.6%	0.981 (0.327; 2.949)	2.819 (0.681; 11.668)

## Data Availability

Raw data will be made available upon request to the corresponding author.

## Supplementary Materials

The following supporting information can be downloaded at https://www.mdpi.com/article/10.3390/v18030274/s1. Supplementary File S1—Strobe checklist.

## Abbreviations

The following abbreviations are used in this manuscript:

ANOVA	Analysis of Variance
aOR	Adjusted Odds Ratio
CoV	Coronavirus
ENT	Enterovirus
hMPV	Human metapneumovirus
ICD	International Classification of Diseases
IE	Immunization effectiveness
INF	Seasonal Influenza Virus
LRTI	Lower respiratory tract infection
mAb	Monoclonal antibody
OR	Odds Ratio
PICU	Pediatric Intensive Care Unit
PIV	Parainfluenza Virus
RIV	Rhinovirus
RR	Risk Ratio
RSV	Respiratory Syncytial Virus
RT-PCR	Real-Time Polymerase Chain Reaction
SARS	Severe-Acute Respiratory Syndrome
SD	Standard Deviation
95% CI	95% Confidence Interval

## Appendix A

**Table A1:** Main pathogens detected from hospitalizations due to lower respiratory tract infections in children and infants (age < 2 years) from participating centers by age groups and months of admissions. In the comparison, RSV was considered as the reference group. Note: RR =. Risk Ratio; 95% CI = 95% Confidence Interval; RSV = human Respiratory Syncytial Virus; RIV/ENT = rhino- and enterovirus; hMPV = human Metapneumovirus.

	RSV	hMPV		RIV/ENT	
	N/190, %	N/29, %	RR (95% CI)	N/81, %	RR (95% CI)
**Age (months)**					
0–2	62, 32.6%	10, 34.5%	1.056 (0.615; 1.816)	46, 56.8%	1.740 (1.317; 2.300)
3–5	44, 23.2%	8, 27.6%	1.191 (0.626; 2.268)	11, 13.6%	0.586 (0.319; 1.076)
6–11	33, 17.4%	5, 17.2%	0.993 (0.422; 2.336)	8, 9.9%	0.569 (0.275; 1.177)
12–24	51, 26.8%	6, 20.7%	0.771 (0.364; 1.632)	16, 19.8%	0.736 (0.447; 1.211)
**Month of admission**					
April to November	19, 10.0%	10, 34.5%	3.448 (1.785; 6.662)	32, 39.5%	3.951 (2.385; 6.543)
December to March	171, 90.0%	19, 65.5%	0.728 (0.557; 0.952)	49, 60.5%	0.672 (0.560; 0.807)

**Table A2:** Summary of main studies on hospitalizations due to lower respiratory tract infections (LRTIs) associated with respiratory syncytial virus (RSV) among Italian children after the implementation of prophylaxis with nirsevimab (note: 95% CI = 95% confidence interval).

Study	RSV LRTI		Non-RSV LRTI		Immunization Effectiveness (Crude, 95% CI)
	Immunized with Nirsevimab	Not immunized with Nirsevimab	Immunized with Nirsevimab	Not Immunized with Nirsevimab	
Attaianese et al. 2025 [46]	19	27	64	28	69.21% (34.55; 85.26)
Manzoni et al. 2025 [41]	49	91	104	65	66.35% (46.28; 78.84)
Cocchi et al. 2025 [81]	9	45	9	22	51.11% (−40.70; 83.05)
Cocchi et al. 2025 [45]	18	15	274	65	71.53% (39.56; 85.95)
Aricò et al. 2025 [82]	12	100	11	44	52.00% (−15.10; 79.36)
Fusco et al. 2025 [40]	20	37	45	19	77.18% (49.60; 88.86)
Fortunato et al. 2025 [112]	13	67	61	111	64.69% (30.66; 82.36)
Maglione et al. 2025 [113]	52	274	35	62	66.38% (43.38; 79.64)
Cumulative (crude estimate)	192	656	603	416	71.04% (63.65; 76.79)
